# Uncovering Factors Related to Pancreatic Beta-Cell Function

**DOI:** 10.1371/journal.pone.0161350

**Published:** 2016-08-18

**Authors:** Aoife M. Curran, Miriam F. Ryan, Elaine Drummond, Eileen R. Gibney, Michael J. Gibney, Helen M. Roche, Lorraine Brennan

**Affiliations:** 1 Institute of Food and Health, School of Agriculture and Food Science, University College Dublin, Dublin, Republic of Ireland; 2 Nutrigenomics Research Group, UCD Conway Institute of Biomolecular and Biomedical Research and UCD Institute of Food and Health, School of Public Health, Physiotherapy and Sports Science, University College Dublin, Belfield, Dublin 4, Republic of Ireland; University of Ulster, UNITED KINGDOM

## Abstract

**Aim:**

The incidence of type 2 diabetes has increased rapidly on a global scale. Beta-cell dysfunction contributes to the overall pathogenesis of type 2 diabetes. However, factors contributing to beta-cell function are not clear. The aims of this study were (i) to identify factors related to pancreatic beta-cell function and (ii) to perform mechanistic studies *in vitro*.

**Methods:**

Three specific measures of beta-cell function were assessed for 110 participants who completed an oral glucose tolerance test as part of the Metabolic Challenge Study. Anthropometric and biochemical parameters were assessed as potential modulators of beta-cell function. Subsequent *in vitro* experiments were performed using the BRIN-BD11 pancreatic beta-cell line. Validation of findings were performed in a second human cohort.

**Results:**

Waist-to-hip ratio was the strongest anthropometric modulator of beta-cell function, with beta-coefficients of -0.33 (p = 0.001) and -0.30 (p = 0.002) for beta-cell function/homeostatic model assessment of insulin resistance (HOMA-IR), and disposition index respectively. Additionally, the resistin-to-adiponectin ratio (RA index) emerged as being strongly associated with beta-cell function, with beta-coefficients of -0.24 (p = 0.038) and -0.25 (p = 0.028) for beta-cell function/HOMA-IR, and disposition index respectively. Similar results were obtained using a third measure for beta-cell function. *In vitro* experiments revealed that the RA index was a potent regulator of acute insulin secretion where a high RA index (20ng ml^-1^ resistin, 5nmol l^-1^ g-adiponectin) significantly decreased insulin secretion whereas a low RA index (10ng ml^-1^ resistin, 10nmol l^-1^ g-adiponectin) significantly increased insulin secretion. The RA index was successfully validated in a second human cohort with beta-coefficients of -0.40 (p = 0.006) and -0.38 (p = 0.008) for beta-cell function/ HOMA-IR, and disposition index respectively.

**Conclusions:**

Waist-to-hip ratio and RA index were identified as significant modulators of beta-cell function. The ability of the RA index to modulate insulin secretion was confirmed in mechanistic studies. Future work should identify strategies to alter the RA index.

## Introduction

The prevalence of type 2 diabetes (T2D) has increased rapidly on an international scale, with pancreatic beta-cell dysfunction and failure at the core of its development [1]. Where hyperglycaemia exists, pancreatic beta-cells must function to a greater capacity in order to produce more insulin to maintain glucose homeostasis [2]. Beta-cells have an ability to functionally adapt to allow for this compensatory response of further insulin production. Beta-cell dysfunction is commonly seen in T2D, where ‘compensation’ of the beta-cells to produce insulin, often due to insulin resistance, leads to the gradual failure of beta-cells [3]. With this in mind, there is a need to investigate factors related to pancreatic beta-cell function in humans.

Glucose stimulates insulin secretion, triggering and amplifying signals in pancreatic beta-cells [4–6]. Challenge tests such as the oral glucose tolerance test (OGTT) have been used to investigate how effective individuals are at maintaining glucose homeostasis, thus assessing beta-cell function [7]. Progression into T2D status can be categorised by examining alterations in metabolic parameters and beta-cell function. Weir & Bonner-Weir proposed five stages of evolving beta-cell dysfunction during the progression into T2D [8]. Stage 1 is described as ‘*compensation’*, where overweight or obese individuals with a degree of insulin resistance have to increase insulin secretion from beta-cells in order to maintain homeostasis. Stage 2 occurs where fasting blood glucose levels range between 5–7.3mmol/L, which represents ‘*beta-cell adaptation’*. Stage 3 represents ‘*early decompensation*’ in which glucose levels rise above 7.3mmol/L, and from this progress rapidly towards a glucose level representative of stage 4, known as ‘*stable decompensation’*, where levels typically range between 16–20mmol/L. Individuals progressing towards T2D can remain in stage 2 for many years, but when beta-cell mass becomes insufficient at an important point, glucose levels rise rapidly to stage 4. Lastly, stage 5 represents ‘*severe decompensation’* and extreme beta-cell failure with advancement to ketosis, with blood glucose levels above 22mmol/L. Movement between stages 1–4 can be in either direction, with diet and exercise interventions having strong potential to return individuals back to stage 2 [8].

It is important to identify parameters which influence the function of beta-cells, in order to optimise beta-cell functionality and potentially identify markers of disease progression or targets for intervention. Body mass index (BMI) and an increased energy intake are recognised as major risk factors for conditions associated with beta-cell dysfunction, and although the evidence of a direct effect of BMI on pancreatic beta-cell function is still largely undefined, the association between BMI and T2D has been well established [9–12]. Strong evidence also exists that an excess of visceral fat is closely related to insulin resistance and T2D risk [13]. The above studies did not have beta-cell dysfunction as their primary aim; therefore further research is needed to determine the exact phenotypic and biochemical parameters that influence specific measures of beta-cell function. A number of recent studies have highlighted a link between beta-cell function and high density lipoprotein (HDL) cholesterol [14–16]. Several studies have found links between certain anthropometric and biochemical parameters associated with T2D, with fewer studies examining the determinants of specific measures of beta-cell function in human cohorts. Beta-cell dysfunction is at the core of T2D, therefore it is paramount to understand factors which influence beta-cell function. In contrast to insulin resistance, beta-cell dysfunction continues to be difficult to measure and monitor, due to factors such as inaccessibility to the endocrine pancreas and incretin effects [17]. There is a clear need for the identification of markers that could be assessed in a fasting biological sample, to allow for the assessment of beta-cell function.

Therefore, the aim of this study was to investigate and identify potential factors related to beta-cell function measures in a human cohort and to further investigate these *in vitro* where possible.

## Materials and Methods

### Study population

This research focuses on data obtained from the Metabolic Challenge (MECHE) study which is part of a national research program by the Joint Irish Nutrigenomics Organisation, as previously described [18]. The MECHE study recruited 214 healthy participants aged between 18–60 years. Individuals were informed about the purpose of the study and the experimental procedures, prior to giving written consent. Good health was defined as the absence of any known chronic or infectious disease and this was verified by a number of fasting blood tests. Details of the study have been published elsewhere [18–21]. Ethical approval was obtained from the Research Ethics Committee at University College Dublin (LS-08-43-Gibney-Ryan) and the study was performed according to the Declaration of Helsinki.

Baseline blood samples were collected on the morning of the study visits following an overnight fast. Participants underwent an OGTT according to the guidelines set by the World Health Organisation/International Diabetes Federation. Venous blood samples were taken before (0 min) and during the OGTT at set time-points (10, 20, 30, 60, 90 and 120 min), and serum and plasma samples were collected as previously described [18–21].

Details of the analytes and methods used are previously reported, along with the measurement of cytokines and hormones [19]. Lipidomic analysis was performed on serum samples (BIOCRATES Life Sciences AG, Innsbruck, Austria), and ceramides were measured using an in-house lipid assay as previously described [18].

For the present study, participants from the MECHE study who underwent an OGTT and who had valid glucose and insulin data at time-points 0 and 30 min were included (n = 110). Their baseline demographic and biochemical parameters were used for analysis. The validation cohort, (Food for Health (FHI) cohort) comprised of 47 healthy overweight and obese participants, with a mean age of 53 years and a mean BMI of 32.1kg m^-2^.

### Measurement of beta-cell function and RA index

Beta-cell function was calculated as the ratio of the incremental insulin to glucose response over the first 30 min of the OGTT (ΔInsulin_30_/ΔGlucose_30_) and three different measures were employed. Firstly, beta-cell function was adjusted for homeostatic model assessment of insulin resistance (HOMA-IR) ((ΔInsulin_30_/ΔGlucose_30_)/HOMAIR). Secondly, the oral disposition index (DI), which takes into account insulin sensitivity, was calculated for all participants $\left(\Delta {Insulin}_{30}/\Delta {Glucose}_{30})\times (\frac{1}{fastingInsulin}\right)$ [22]. Thirdly, beta-cell function was calculated and adjusted for the Matsuda Index 10000/√(Glucose_0_ × Insulin_0_ × Glcuose_120_ × Insulin_120_) (, where glucose is in mg dl^-1^and insulin in iU ml^-1^[23]. Additionally, C-peptide data was substituted for insulin data for the DI, for the beta-cell function (ΔCpeptide_30_/ΔGlucose_30_), and beta-cell function adjusted for the Matsuda Index (ΔCpeptide_30_/ΔGlucose_30_) × Matsuda Index,.

A ratio of resistin (R_0_) to adiponectin (A_0_) (RA index) was formulated as follows:

$$RA\;index=R_{0}/A_{0}$$

Where: R_0_ = fasting plasma resistin levels (ng ml^-1^) and A_0_ = fasting serum total adiponectin levels (μg ml^-1^)

### Cell culture and treatment

All chemicals were purchased from Sigma-Aldrich Ireland unless otherwise stated. Culture media and its related components were purchased from Gibco (Glasgow, UK). The BRIN-BD11 cell line was used in this study [24] and was maintained as previously described [25].

For experimental treatments, cells were seeded at a density of 1.5 x 10^5^ cells per well in a 24 well plate for insulin secretion assays. Cells were allowed to attach for 24 h before being treated with recombinant rat resistin (Cambridge Biosciences, Cambridge, UK) or rat GACRP30/Adiponectin (Sigma-Aldrich) or ratios of both, for 24 h. Concentrations of 10–20ng ml^-1^ of resistin and 5–20nmol l^-1^ of globular (g) adiponectin were used. Concentrations were chosen in accordance with previous studies [26, 27]. Cells between passage 23–33 were used and all experiments were n = 4 unless otherwise stated.

### Acute insulin secretion

Following the 24 h treatment period, the culture medium was removed and the cells were washed with phosphate buffered saline (PBS). The cells were then incubated with Krebs-Ringer bicarbonate (KRB) buffer (115mM NaCL, 1.28mM CaCl_2_, 4.7mM KCl, 1.2mM KH_2_PO_4_, 1.2mM MgSO_4_ 7H_2_O, 10mM NaHC0_3,_ 5 g l^-1^ BSA, all at pH 7.4) supplemented with 1.1mM glucose for 40 min. The media was then replaced with KRB buffer containing 16.7mM glucose + 10mM alanine, for 20 min. Following this, the samples were transferred to Eppendorfs and centrifuged, before removing the supernatant and assaying for insulin content using a Mercodia Ultrasensitive Rat Insulin ELISA kit (Mercodia AB, Uppsala, Sweden).

### Measurement of mitochondrial membrane potential, intracellular calcium and plasma membrane potential

In order to measure mitochondrial membrane potential, a protocol based on Rhodamine fluorescence as described by Wallace *et al* was followed [25]. Cells were treated for 24 h with high and low RA index. Fluorescence was measured over a period of 150 seconds collecting data every 3 seconds, with injection of glucose to a final concentration of 16.7mM + 10mM alanine at 50 seconds.

Intracellular calcium was analysed using the FLIPR Calcium 4 assay kit (R8141 Bulk Kit Molecular Devices), as described by Wallace *et al* [25]. Cells were treated for 24 h with high and low RA index. Fluorescence was then measured in a Flexstation, with readings every 2.5 seconds for 10 min. Cells were stimulated at 100 seconds with 16.7mM glucose + 10mM alanine).

Plasma membrane potential was also determined. Following 24 hour treatment with a high and low RA index, media was removed and the cells were incubated with 100μl of 2.2mM glucose KRB buffer and 100μl of loading dye (FLIPR blue membrane potential buffer (Molecular Devices)) for 20 min. Fluorimetric data was acquired on the Flexstation with an excitation wavelength of 530nm and an emission wavelength of 565nm. The Flexstation was set to run for 350 seconds, collecting data at 2.5 second intervals, with stimulation of the cells (16.7mM glucose + 10mM alanine) occurring at 100 seconds.

### Gene expression analysis

Cells were seeded in 6 well plates and allowed to reach 80% confluence before treatment with high RA index and low RA index for 24 h. Total RNA was extracted using TRIzol reagent (Invitrogen). Reverse transcription of 2 μg of total RNA was carried out using random primers and SuperScript II (Invitrogen by Life Technologies). Samples were incubated in a PCR incubator for 25°C for 10 min, 42°C for 50 min and 70°C for 15 min. The expression of *Pancreatic and duodenal homeobox 1 (PDX1)*, *Insulin receptor (INSR)*, *Adiponectin receptor 1 (ADIPOR1)* and *Adiponectin receptor 2 (ADIPOR2)* were investigated by real time PCR on an Applied Biosystems 7900HT fast real-time PCR system using TaqMan gene-specific assays (*PDX1* (assay Rn00755591_m1), *INSR* (assay Rn00690703_m1), *ADIPOR1* (assay Rn01483784_m1) and *ADIPOR2* (assay Rn01463173_m1)). The results were normalised to beta-actin and cyclophilin A expression.

### Statistical Analysis

Analysis was carried out using IBM SPSS Statistics V.20. Data are expressed as means ± standard deviation. Linear regression analysis was carried out to examine relationships between beta-cell function and various anthropometric and biochemical parameters. Statistical significance was evaluated using ANOVA with LSD and Bonferroni post-hoc tests. Significant differences were observed if P ≤ 0.05. For gene expression analysis, primary analysis was carried out using Sequence Detection Software (SDS) 2.4, and secondary analysis used the software package Data Assist 3.01.

## Results

### Study population

Analysis was performed on a total of 110 participants who underwent an OGTT. Baseline characteristics are presented in Table 1. An equal gender balance existed with 55 males and 55 females. The mean body mass index was 25.3kg m^-2^, which lies at the lower end of the overweight BMI category (25.0–29.9kg m^-2^).

**Table 1:** Baseline characteristics of MECHE cohort (n = 110).

Variable	Mean ± S.D.
**Sex (m/f)**	55/55
**Age (y)**	32 ± 11
**Weight (kg)**	76.65 ± 16.85
**BMI (kg m^-2^)**	25.3 ± 5.3
**WHR**	0.85 ± 0.1
**BP SYS (mm Hg^-1^)**	123.1 ± 12.9
**BP DIA (mm Hg^-1^)**	74.7 ± 10.9
**Glucose (mmol l^-1^)**	5.21 ± 0.56
**HDL cholesterol (mmol l^-1^)**	1.34 ± 0.36
**TAG (mmol l^-1^)**	1.05 ± 0.60
**Insulin (μIU ml^-1^)**	8.48 ± 6.69
**HOMA-IR**	2.00 ± 1.70
**Adiponectin (ug ml^-1^)**	4.99 ± 3.07
**Resistin (ng ml^-1^)**	4.56 ± 1.77

All values are means ± standard deviation. BMI, Body Mass Index; WHR, Waist to Hip Ratio; BP SYS, Systolic Blood Pressure; BP DIA, Diastolic Blood Pressure; HDL, High Density Lipoprotein cholesterol; TAG, triglycerides; HOMA-IR, Homeostatic Model Assessment of Insulin Resistance

### Identification of factors related to beta-cell function

Gender had no significant relationship with beta-cell function measures. Investigation into the effect of BMI revealed that as BMI increased, beta-cell function decreased (Table 2). As described in Table 3 waist-to-hip ratio was the strongest predictor of beta-cell function, with beta-coefficients of -0.33, -0.30, and -0.26 for beta-cell function/HOMA-IR, DI and beta-cell function adjusted for Matsuda index respectively. Further examination of the biochemical parameters revealed that the RA index had a strong relationship with beta-cell function with beta-coefficients of -0.24–0.25, and -0.25 for beta-cell function/HOMA-IR, DI and beta-cell function adjusted for Matsuda index respectively. C12:1(2H) was the strongest predictor of beta-cell function when ceramide data was examined. The list of ceramides analysed are present in S1 Table. Additionally, when C-peptide was used to calculate the DI and beta-cell function adjusted for the Matsuda index similar results emerged (S2 Table).

**Table 2:** Beta-cell function, resistin and adiponectin according to BMI categories.

BMI Categories (kg m^-2^)			
	Group 1 (18–24.9 kg m^-2^)	Group 2 (>25 kg m^-2^)	*P*
	(n = 60)	(n = 46)	
**Beta-cell function/ HOMA-IR (pmol mmol**^**-1**^**)**	14.26 ± 10.61	9.55 ± 6.98	0.04
**Disposition index (pmol mmol**^**-1**^**)**	3.22 ± 2.21	2.48 ± 1.78	0.07
Beta-cell function* **Matsuda index**	14.50 ±13.38	10.37 ± 11.94	0.11
**Disposition index (C-Peptide) (nmol mmol**^**-1**^**)**	3.07 ± 1.97	2.31 ± 2.26	0.07
Beta-cell function (C-peptide)* **Matsuda index**	24.17 ± 20.06	16.71 ± 16.77	0.05
**Resistin (ng ml**^**-1**^**)**	4.31 ± 1.54	4.70 ± 1.98	0.25
**Adiponectin (μg ml**^**-1**^**)**	5.73 ± 3.12	3.55 ± 2.28	<0.001

All values are means ± standard deviation. P-value determined using independent samples t-test (Significance level (P = <0.05)).

* indicates multiplication

**Table 3:** Linear regression of anthropometric, biochemical and ceramide data against beta-cell function measures.

Predictor	Beta-cell function/ HOMA-IR (pmol mmol^-1^)		Disposition index (pmol mmol^-1^)		Beta-cell function* Matsuda index	
	Beta coefficient	*P*	Beta coefficient	*P*	Beta coefficient	*P*
**WHR**	-0.33	0.001	-0.30	0.002	-0.26	0.016
**RA index**	-0.24	0.038	-0.25	0.028	-0.25	0.021
**Cer 12:1(2H)**	-0.24	0.015	-0.24	0.021	-0.23	0.010

Summary of strongest predictors of beta-cell function using linear regression analysis. WHR, waist-to-hip ratio; HDL, high density lipoprotein cholesterol; RA index, resistin-to-adiponectin ratio; cer, ceramide. Data are presented as beta coefficient and P-value according to beta-cell function/HOMA-IR; Homeostatic Model Assessment of Insulin Resistance and DI; Disposition index; beta-cell function (glucose in mg dl^-1^, insulin in μIU ml^-1^) adjusted for the Matsuda index; P-value determined using backward linear regression analysis. Significance level = P < 0.05. Demographic and Anthropometric variables included were: age, sex, BMI, WHR, BP SYS, BP DIA. Biochemical variables included were: HDL cholesterol, adiponectin, resistin, RA index, triacylglycerides, Apo E, TNFα, IFNγ, IL2, IL4, IL6, IL8, IL10. Ceramide data from lipidomic analysis was examined.

* indicates multiplication.

To validate the relationship between the RA index and beta-cell function, adiponectin and resistin concentrations were measured in a second cohort. This cohort, the Food for Health (FHI) cohort comprised of 47 healthy overweight and obese, slightly older participants and had a mean age of 53 years and a mean BMI of 32.1kg m^-2^. The RA index was successfully validated in this second human cohort with beta coefficients of -0.395 (p = 0.006), -0.384 (p = 0.008) and -0.540 (p<0.0001) for beta-cell function/ HOMA-IR, DI and beta-cell function adjusted for Matsuda index respectively (S3 Table).

### RA index modulates insulin secretion in pancreatic beta-cell line

**Fig 1:**
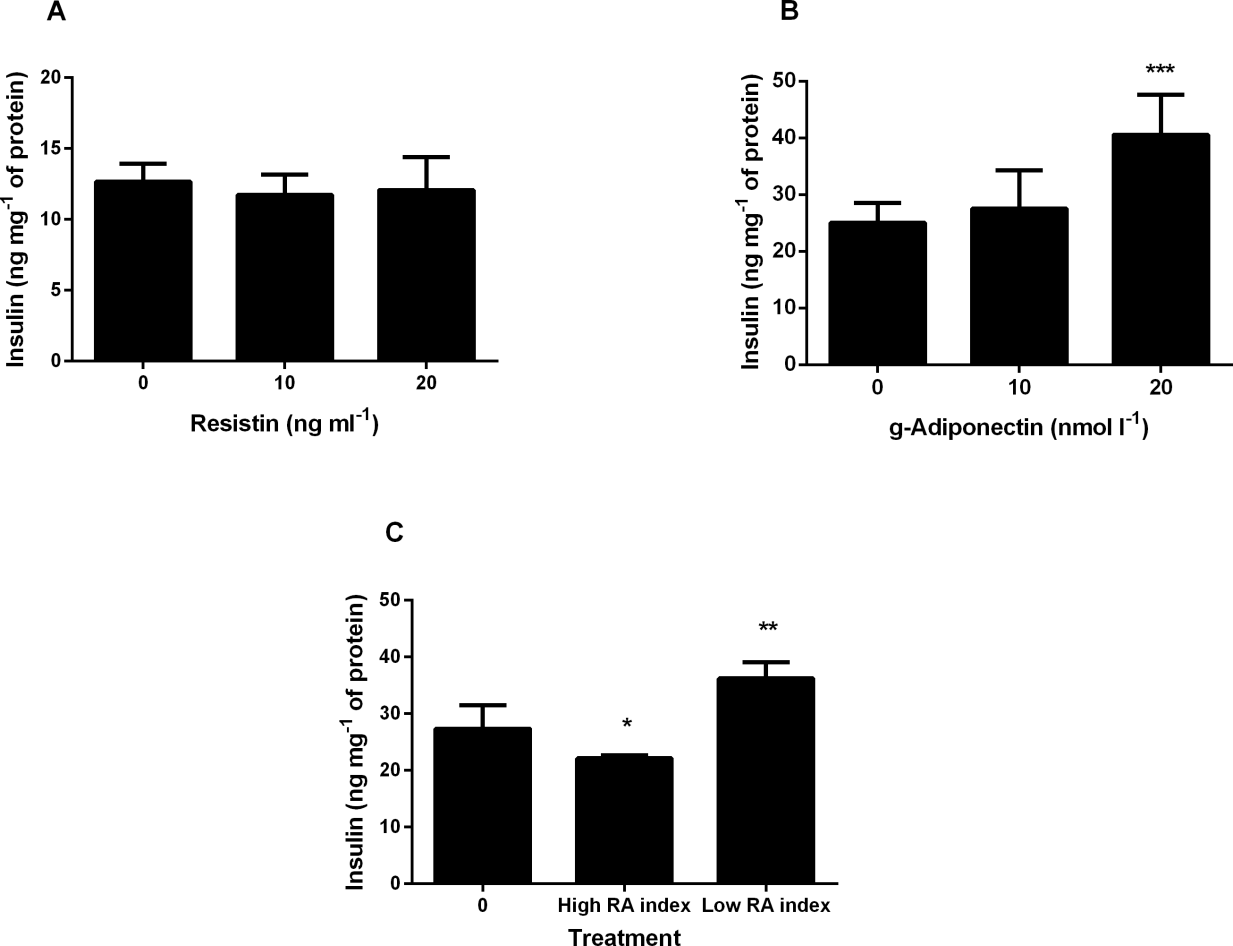
The effect of 24 hour treatment with resistin, g-adiponectin, or both (representing different RA indices) on insulin secretion in BRIN-BD11 cell line. Values are mean ± standard deviation (n = 4). *p < 0.05 **p < 0.01 *** p < 0.001. ANOVA was applied across groups with post-hoc LSD test for comparison of resistin, g-adiponectin, and high and low RA index with no treatment (control). **(A)** Cells were incubated for 24 h with 0, 10 and 20ng ml^-1^ resistin and then stimulated with 16.7mM glucose + 10mM alanine to determine insulin secretion. **(B)** Cells were incubated for 24 h with 0, 10 and 20nmol l^-1^ g-adiponectin, and then stimulated with 16.7mM glucose + 10mM alanine to determine insulin secretion. Overall p-value = 0.00003. **(C)** Cells were incubated for 24 h with no treatment (control), high RA index (20ng ml^-1^ resistin, 5nmol l^-1^ g-adiponectin) and a low RA index (10ng ml^-1^ resistin, 10nmol l^-1^ g- adiponectin) and then stimulated with 16.7mM glucose + 10mM alanine to determine insulin secretion. Overall p-value = 0.0003.

To assess the effects of exposure to resistin, g-adiponectin or a ratio of both, on pancreatic beta-cells, BRIN-BD11 cells were incubated with the adipokines for 24 h. There was no loss of cell viability during the incubation period. Following exposure to resistin no significant effect on insulin secretion was observed (Fig 1A). Conversely exposure to g-adiponectin resulted in a significant increase in insulin secretion at the higher concentration of 20nmol l^-1^ g-adiponectin (Fig 1B). A dose response study of various RA indexes was carried out (S1 Fig). From this dose response data we chose the RA indexes that elicited the lowest and highest insulin secretion response. Following treatment with two different RA indexes, a high RA index (20ng ml^-1^ resistin, 5nmol l^-1^ g-adiponectin) significantly decreased insulin secretion whereas a low RA index (10ng ml^-1^ resistin, 10nmol l^-1^ g-adiponectin) significantly increased insulin secretion (Fig 1C). Interestingly at this concentration of adiponectin alone there was no significant increase in insulin secretion indicating the importance of the ratio.

**Fig 2:**
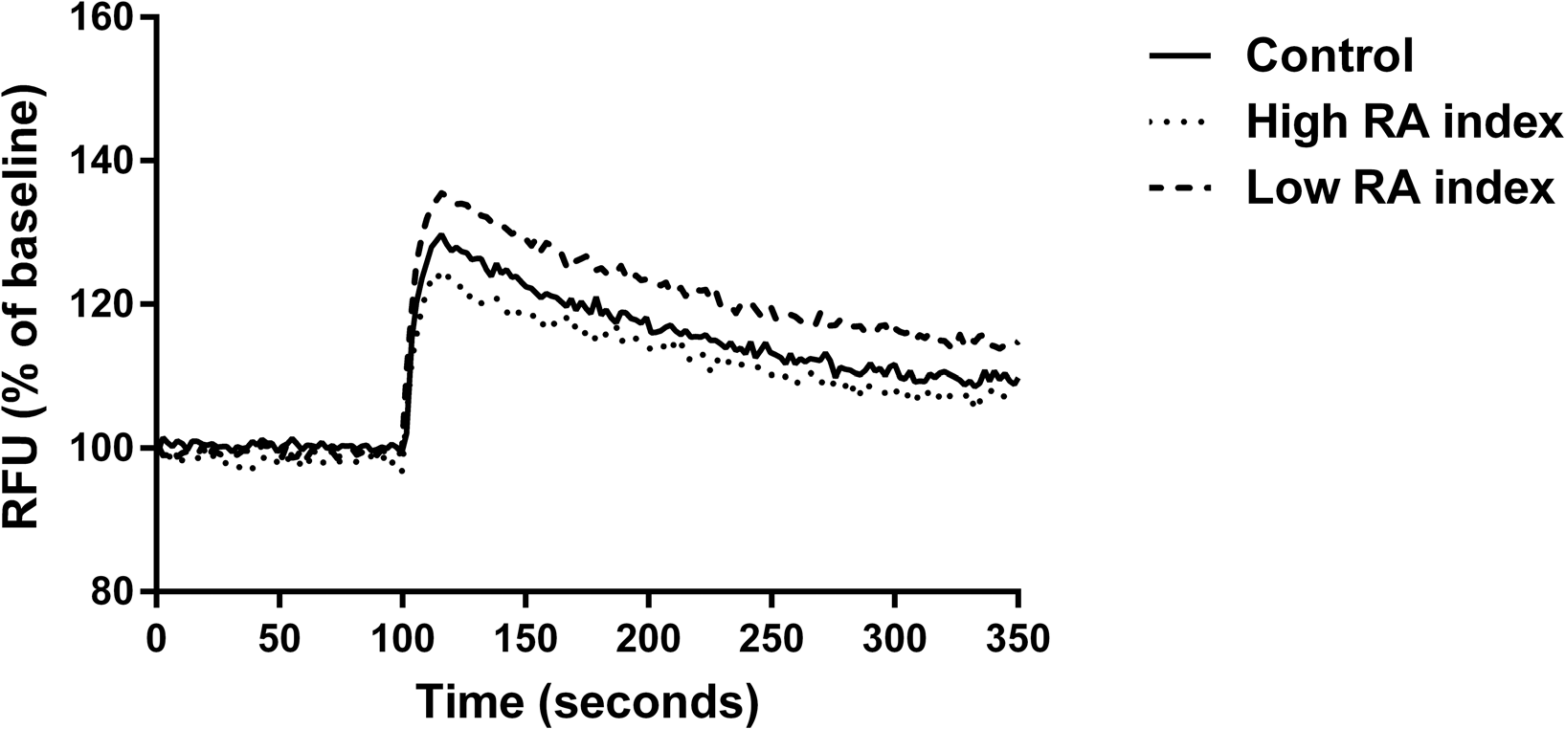
The effect of RA index on the plasma membrane potential. BRIN-BD11 cells were treated for 24 h with a control (no treatment), high RA index (20ng ml^-1^ resistin, 5nmol l^-1^ g-adiponectin) and a low RA index (10ng ml^-1^ resistin, 10nmol l^-1^ g-adiponectin). Cells were stimulated with 16.7mM glucose + 10mM alanine at 100 seconds. Data was analysed by determining the difference in relative fluorescence units (RFU) between the average baseline and post stimulation values for each experiment (delta change %). The increase in fluorescence (normalised to baseline) upon stimulation was 26.4% for control, 23.5% for high RA index and 33.9% for low RA index. Statistically significant differences exist upon the increase in RFU between control treatment and low RA index (p = 0.009) and high and low RA index (p = 0.003). Overall ANOVA p = 0.007. Values are represented as mean values (n = 5).

Functional assays revealed that plasma membrane potential of cells treated with the low RA index was significantly greater in comparison to the control (no treatment). The high RA index displayed significantly lower plasma membrane potential compared to the low RA index (Fig 2). No significant differences were seen between treatments in mitochondrial membrane assays or intracellular calcium assays (S2 and S3 Figs).

**Fig 3:**
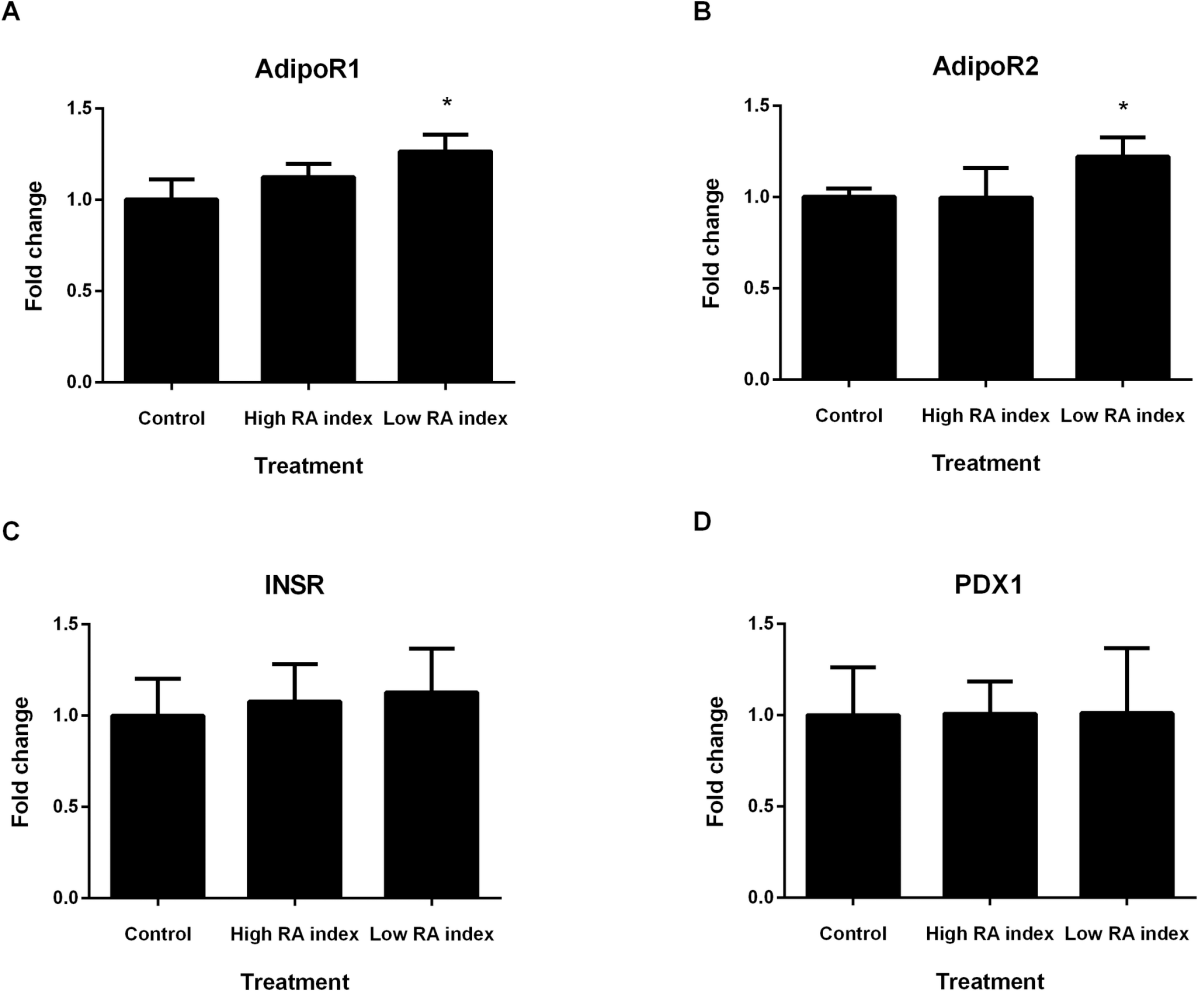
Gene expression analysis of BRIN-BD11 cells treated with RA index. Low RA index significantly increases (**A)** *ADIPOR1* and (**B)** *ADIPOR2* mRNA expression in BRIN-BD11 cells. (**C)** No effect on *INSR* expression was observed when cells were treated with high and low RA index. (**D)** *PDX1* expression was not altered by high or low RA index treatment. Experiments n = 6, *p < 0.05 versus the respective control.

Real time PCR analysis revealed significant increases in expression of *ADIPOR1* and *ADIPOR2* when cells were treated with low RA index. Treatment with the different RA indexes did not impact on gene expression levels of *PDX1* and *INSR* (Fig 3).

## Discussion

The RA index and waist-to-hip ratio were revealed to be strongly associated with pancreatic beta-cell function. The *in vitro* studies support the relationship between the RA index and beta-cell function in terms of insulin secretion. Although previous results have shown the ratio to be a predictor of T2D development, to the best of our knowledge this is the first to report a direct relationship with pancreatic beta-cell function.

Dysregulation of adipokine secretion is frequently observed in obesity and T2D [28]. Circulating adiponectin in humans typically ranges between 2–30μg ml^-1^, while the serum concentration of resistin ranges from 7 to 22 ng ml^-1^ [29, 30]. Adiponectin has been associated with insulin sensitivity and metabolism of lipids in peripheral tissues [31, 32], along with stimulating insulin secretion [33]. Furthermore adiponectin has also been found to exert cytoprotective effects in beta-cells in vivo, and aids in protecting cells from undergoing apoptosis [27]. Resistin has been associated with insulin resistance and pro-inflammatory properties, along with impaired insulin secretion, and is believed to be an important link between obesity, insulin resistance and T2D [34, 35]. Resistin treatment impedes glucose tolerance and insulin response in mouse models [36], and modulates cell viability in cell lines [26]. However the translation of these findings to humans has been less conclusive, with mixed findings emerging [37, 38].

In support of our results a previous study identified that a resistin to adiponectin ratio was associated with T2D and Metabolic Syndrome (MS) risk [39]. Moreover, this study demonstrated that the ratio of resistin to adiponectin was more strongly correlated with insulin resistance indexes and key metabolic endpoints of T2D and MS than adiponectin and resistin levels alone. This together with our data support the role of the RA index as a potential biomarker of beta-cell function status; use of such a biomarker profile to identify persons at risk of development of T2D could be an important step in the development of targeted lifestyle interventions. Accurate assessment of beta-cell function from a fasting blood sample would allow for earlier identification of beta-cell dysfunction and make it easier to monitor an individual’s risk of progression into T2D.

Although the present cohort was generally healthy, 46 participants fell into an overweight and obese BMI category (>25kg m^-2^). Analysis between normal BMI and overweight and obese BMI categories revealed a significant decrease in both beta-cell function/HOMA-IR and DI as BMI increased. Importantly, an intervention study in 11 obese T2D individuals revealed that reducing BMI through energy restriction (600kcal/day) for 8 weeks resulted in significant improvements in beta-cell function [40]. A significant decrease in waist circumference (107.4 ± 2.2cm at baseline to 94.2 ± 2.5cm at week 8) was also observed in the intervention. Based on the present analysis, waist-to-hip ratio was a strong modulator of beta-cell function, when demographic and anthropometric variables were examined. Waist-to-hip ratio emerged as a stronger modulator of beta-cell function than BMI, which is interesting as it therefore may be a better indicator of T2D risk than a BMI score. Supporting evidence for this exists in the literature where waist-to-hip ratio was determined to be a stronger predictor than BMI of T2D risk in a small Taiwanese cohort [41]. This finding also adds to the hypothesis that central obesity and body shape may be important considerations when in assessing T2D risk, due to strong evidence that an excess of visceral fat is closely related to insulin resistance and T2D risk [13]. In a study by Bardini *et al*. (2011), a hypertriglyceridaemic waist phenotype (enlarged waist circumference and increased triglyceride levels) was associated with increased insulin resistance and an overexertion of beta-cell function in participants with normal glucose tolerance, while participants with impaired glucose tolerance and a hypertriglyceridaemic waist phenotype displayed a decrease in beta-cell function. This highlights the importance of implementing an early intervention to decrease T2D risk [42]. Ceramide 12:1(2H) was also predictive of beta-cell function in our cohort. Ceramides are suggested to be responsible for beta-cell apoptosis due to saturated fatty acid exposure, however the mechanism behind how ceramide accumulation leads to this is still unclear [43].

*In vitro* verification of the improved beta-cell functionality is an important aspect of this study: the low RA index significantly modulated acute insulin secretion. Functional assays revealed that there was a significant increase in plasma membrane potential in cells treated with the low RA index: this enhancement could underpin the increased insulin secretion under these conditions. Previous studies have examined alteration of plasma membrane potential of cells treated with adiponectin, with mixed findings. A study examining adiponectin treatment in pancreatic islets found no effects on membrane potential, however another study by Wen *et al* investigating adiponectin treatment in hypothalamic cells observed plasma membrane hyperpolarisation [44, 45]. In addition to alterations in the plasma membrane potential significant increases in *ADIPOR1* and *ADIPOR2* expression were observed following treatment with the low RA index. Adiponectin acts by binding and activating *ADIPOR1* and *ADIPOR2*, and the increased expression of both receptors with low RA index treatment suggests that it plays a role in the regulation of beta-cell function [46, 47]. This increase in adiponectin receptor expression in conjunction with the alterations in plasma membrane potential provides a potential mechanism for the promotion of insulin secretion under these conditions.

Strengths of the present study include directly assessing factors related to specific beta-cell measures obtained during an OGTT and confirmation in an independent cohort. *In vitro* results mirrored the findings in the human studies and provided an opportunity to examine potential mechanisms by which the RA index promoted insulin secretion. Validation of the RA index in the FHI human cohort, a cohort slightly older and with a greater BMI than the MECHE cohort, also strengthens the case of the RA index as a factor related to beta-cell function. The present study population is limited to Irish participants and it is acknowledged that expansion of this research to non-Irish and non-European cohorts would be beneficial in order to fully translate the research findings to the global population.

## Conclusions

In conclusion, our findings indicate that waist-to-hip ratio and RA index are strong factors related to pancreatic beta-cell function. Establishing whether alterations in the RA index is a causative factor in development of T2D is a question which remains to be answered. Furthermore, investigation of the ability to modify the RA index through lifestyle interventions will be key to the potential use of such an index. Future work will examine potential mechanisms for modulating the RA index which in turn may lead to new routes/interventions for improving beta-cell function.

## Supporting Information

S1 Fig The effect of 24 hour treatment with different RA indices on insulin secretion in BRIN-BD11 cell line. Values are mean ± standard deviation (n = 4). *p < 0.05 **p < 0.01 *** p < 0.001. ANOVA was applied across groups with post-hoc LSD test for comparison of various RA indexes with no treatment (control). Cells were incubated for 24 hours with no treatment (control), 0.1 ratio (5ng ml^-1^ resistin and 50nmol l^-1^ g-adiponectin), 0.5 ratio (10ng ml^-1^ resistin and 20nmol l^-1^ g-adiponectin), 1.0 ratio (10ng ml^-1^ resistin, 10nmol l^-1^ g- adiponectin), 2.0 ratio (20ng ml^-1^ resistin, 10nmol l^-1^ g- adiponectin) 4.0 ratio (20ng ml^-1^ resistin, 5nmol l^-1^ g-adiponectin) and then stimulated with 16.7mM glucose + 10mM alanine to determine insulin secretion. Overall p-value = 0.000053
(DOCX)

S2 Fig The effect of RA index on changes in mitochondrial membrane potential. BRIN-BD11 cells were treated for 24 h with a control (no treatment), high RA index (20ng ml^-1^ resistin, 5nmol l^-1^ g-adiponectin) and a low RA index (10ng ml^-1^ resistin, 10nmol l^-1^ g-adiponectin). Cells were stimulated with 16.7mM glucose + 10mM alanine at 50 seconds and mitochondrial membrane potential was assessed. Data was analysed by determining the difference in relative fluorescence units (RFU) between the average baseline and post stimulation values for each experiment (delta change %). The decrease in fluorescence (normalised to baseline) upon stimulation was 18.9% for control, 21.8% for high RA index and 20.7% for low RA index. No statistically significant differences exist upon the decrease in RFU between control treatment and high and low RA index (overall ANOVA p = 0.758). Values are represented as mean values (n = 4).
(DOCX)

S3 Fig The effect of RA index on changes on intracellular calcium. BRIN-BD11 cells were treated for 24 h with a control (no treatment), high RA index (20ng ml^-1^ resistin, 5nmol l^-1^ g-adiponectin) and a low RA index (10ng ml^-1^ resistin, 10nmol l^-1^ g-adiponectin). Cells were stimulated with 16.7mM glucose + 10mM alanine at 100 seconds and intracellular calcium was assessed. Data was analysed by determining the difference in relative fluorescence units (RFU) between the average baseline and post stimulation values for each experiment (delta change %). The increase in fluorescence (normalised to baseline) upon stimulation was 44.3% for control, 40.2% for high RA index and 46.1% for low RA index. No statistically significant differences exist upon the increase in RFU between control treatment and high and low RA index (overall ANOVA p = 0.728). Values are represented as mean values (n = 4).
(DOCX)

S1 Table List of ceramides from MECHE lipidomic dataset. CER: ceramide. List of ceramides measured in MECHE serum samples.
(DOCX)

S2 Table Linear regression of anthropometric, biochemical and ceramide data against additional beta-cell function measures. Summary of strongest predictors of beta-cell function using linear regression analysis. WHR, waist-to-hip ratio; HDL, high density lipoprotein cholesterol; RA index, resistin-to-adiponectin ratio; cer, ceramide. Data are presented as beta coefficient and P-value according to disposition index, using C-peptide data (nmol mmol^-1^); beta-cell function using C-peptide (glucose in mmol l^-1^, c-peptide in nmol l^-1^) adjusted for the Matsuda index; P-value determined using backward linear regression analysis. Significance level = P < 0.05. Demographic and Anthropometric variables included were: age, sex, BMI, WHR, BP SYS, BP DIA. Biochemical variables included were: HDL cholesterol, adiponectin, resistin, RA index, triacylglycerides, Apo E, TNFα, IFNγ, IL2, IL4, IL6, IL8, IL10. Ceramide data from lipidomic analysis was examined. *RA index in combination with IL-8 was significant predictor of beta-cell function (C-peptide)* Matsuda index using linear regression (p = 0.043).
(DOCX)

S3 Table Baseline characteristics FHI cohort (n = 47). All values are means ± standard deviation. BMI, Body Mass Index; BP SYS, Systolic Blood Pressure; BP DIA, Diastolic Blood Pressure; HOMA-IR, Homeostatic Model Assessment of Insulin Resistance; BCF/HOMA-IR, beta-cell function adjusted by HOMA-IR; BCF*Matsuda index; beta-cell function adjusted by the Matsuda index (where glucose mg dl^-1^and insulin μIU ml^-1^) RA index, resistin to adiponectin ratio.
(DOCX)
