# Supplementary material for: Uncovering Factors Related to Pancreatic Beta-Cell Function
Source: PLoS One. 2016 Aug 18;11(8):e0161350. doi: 10.1371/journal.pone.0161350 (PMC4990237; doi:10.1371/journal.pone.0161350)
Supplement: S1 Fig — Values are mean ± standard deviation (n = 4). *p < 0.05 **p < 0.01 *** p < 0.001. ANOVA was applied across groups with post-hoc LSD test for comparison of various RA indexes with no treatment (control). Cells were incubated for 24 hours with no treatment (control), 0.1 ratio (5ng ml-1 resistin and 50nmol l-1 g-adiponectin), 0.5 ratio (10ng ml-1 resistin and 20nmol l-1 g-adiponectin), 1.0 ratio (10ng ml-1 resistin, 10nmol l-1 g- adiponectin), 2.0 ratio (20ng ml-1 resistin, 10nmol l-1 g- adiponectin) 4.0 ratio (20ng ml-1 resistin, 5nmol l-1 g-adiponectin) and then stimulated with 16.7mM glucose + 10mM alanine to determine insulin secretion. Overall p-value = 0.000053 (DOCX) [file pone.0161350.s001.docx]

**Online Supplementary Material**


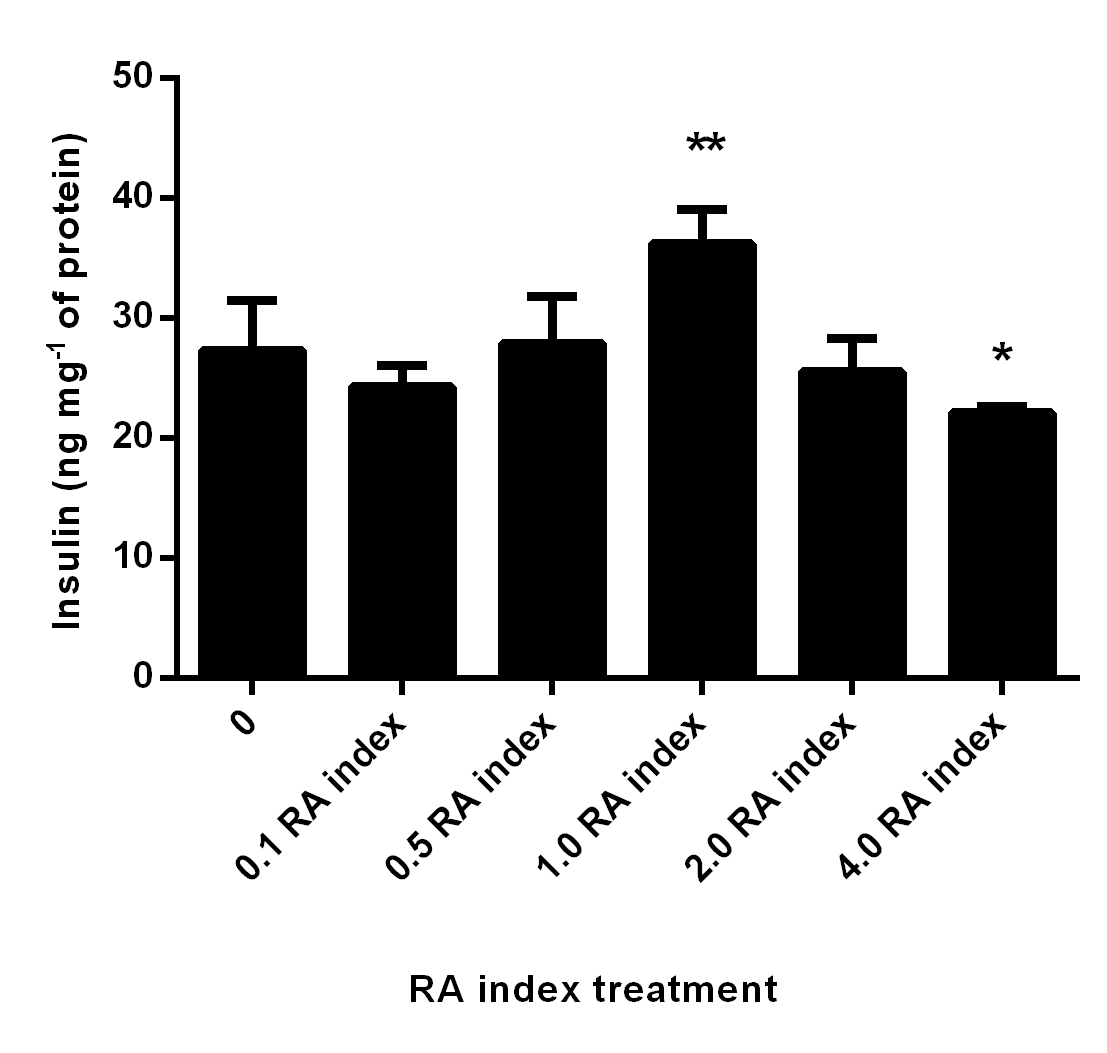


**S1 Fig. The effect of 24 hour treatment with different RA indices on insulin secretion in BRIN-BD11 cell line.**

Values are mean ± standard deviation (n = 4). *p < 0.05 **p < 0.01 *** p < 0.001. ANOVA was applied across groups with post-hoc LSD test for comparison of various RA indexes with no treatment (control).

Cells were incubated for 24 h with no treatment (control), 0.1 ratio (5ng ml^-1^ resistin and 50nmol l^-1^ g-adiponectin), 0.5 ratio (10ng ml^-1^ resistin and 20nmol l^-1^ g-adiponectin), 1.0 ratio (10ng ml^-1^ resistin, 10nmol l^-1^ g- adiponectin), 2.0 ratio (20ng ml^-1^ resistin, 10nmol l^-1^ g- adiponectin) 4.0 ratio (20ng ml^-1^ resistin, 5nmol l^-1^ g-adiponectin) and then stimulated with 16.7mM glucose + 10mM alanine to determine insulin secretion. Overall p-value = 0.000053
