# Supplementary material for: Uncovering Factors Related to Pancreatic Beta-Cell Function
Source: PLoS One. 2016 Aug 18;11(8):e0161350. doi: 10.1371/journal.pone.0161350 (PMC4990237; doi:10.1371/journal.pone.0161350)
Supplement: S3 Fig — BRIN-BD11 cells were treated for 24 h with a control (no treatment), high RA index (20ng ml-1 resistin, 5nmol l-1 g-adiponectin) and a low RA index (10ng ml-1 resistin, 10nmol l-1 g-adiponectin). Cells were stimulated with 16.7mM glucose + 10mM alanine at 100 seconds and intracellular calcium was assessed. Data was analysed by determining the difference in relative fluorescence units (RFU) between the average baseline and post stimulation values for each experiment (delta change %). The increase in fluorescence (normalised to baseline) upon stimulation was 44.3% for control, 40.2% for high RA index and 46.1% for low RA index. No statistically significant differences exist upon the increase in RFU between control treatment and high and low RA index (overall ANOVA p = 0.728). Values are represented as mean values (n = 4). (DOCX) [file pone.0161350.s003.docx]

**Online Supplementary Material**





**S3 Fig**. **The effect of RA index on changes on intracellular calcium.**

BRIN-BD11 cells were treated for 24 h. with a control (no treatment), high RA index (20ng ml^-1^ resistin, 5nmol l^-1^ g-adiponectin) and a low RA index (10ng ml^-1^ resistin, 10nmol l^-1^ g-adiponectin). Cells were stimulated with 16.7mM glucose + 10mM alanine at 100 seconds and intracellular calcium was assessed. Data was analysed by determining the difference in relative fluorescence units (RFU) between the average baseline and post stimulation values for each experiment (delta change %). The increase in fluorescence (normalised to baseline) upon stimulation was 44.3% for control, 40.2% for high RA index and 46.1% for low RA index. No statistically significant differences exist upon the increase in RFU between control treatment and high and low RA index (overall ANOVA p = 0.728). Values are represented as mean values (n=4).
