# Supplementary material for: Uncovering Factors Related to Pancreatic Beta-Cell Function
Source: PLoS One. 2016 Aug 18;11(8):e0161350. doi: 10.1371/journal.pone.0161350 (PMC4990237; doi:10.1371/journal.pone.0161350)
Supplement: S1 Table — CER: ceramide. List of ceramides measured in MECHE serum samples. (DOCX) [file pone.0161350.s004.docx]

**Online Supplementary Material**

**S1 Table. List of ceramides from MECHE lipidomic dataset**

| CER 7:0(OH) | CER 14:0 | CER 19:0(OH)(2H) | CER 24:0(OH) |
| --- | --- | --- | --- |
| CER 7:0(OH)(2H) | CER 14:0(2H) | CER 19:0 | CER 24:0 (OH)(2H) |
| CER 7:0 | CER 14:1(2H) | CER 19:1 | CER 24:0 |
| CER 7:0(2H) | CER 15:0(OH) | CER 19:1(2H) | CER 24:0(2H) |
| CER 7:1 | CER 15:0(OH)(2H) | CER 20:0(OH) | CER 24:1 |
| CER 7:1(2H) | CER 15:0 | CER 20:0(OH)(2H) | CER 24:1(2H) |
| CER 8:0(OH) | CER 15:0(2H) | CER 20:0 | CER 25:0(OH) |
| CER 8:0 (OH)(2H) | CER 15:1(2H) | CER 20:0(2H) | CER 25:0 (OH)(2H) |
| CER 8:0 | CER 16:0(OH) | CER 20:1 | CER 25:0 |
| CER 8:0(2H) | CER 16:0(OH)(2H) | CER 20:1(2H) | CER 25:0(2H) |
| CER 9:0(OH) | CER 16:0 | CER 21:0(OH) | CER 25:1 |
| CER 9:1 | CER 16:0(2H) | CER 21:0(OH)(2H) | CER 25:1(2H) |
| CER 10:0 (OH) | CER 16:1 | CER 21:0 | CER 26:0(OH) |
| CER 10:0(OH)(2H) | CER 16:1(2H) | CER 21:0(2H) | CER 26:0(OH)(2H) |
| CER 10:0 | CER 17:0(OH) | CER 22:0(OH) | CER 26:0 |
| CER 11:0(OH) | CER 17:0(OH)(2H) | CER 22:0(OH)(2H) | CER 26:0(2H) |
| CER 11:0(OH)(2H) | CER 17:0 | CER 22:0 | CER 26:1 |
| CER 11:0 | CER 17:0(2H) | CER 22:0(2H) | CER 27:0 |
| CER 11:1 | CER 17:1(2H) | CER 22:1 | CER 27:1 |
| CER 12: 0(OH) | CER 18:0(OH) | CER 22:1(2H) | CER 28:0(OH)(2H) |
| CER 12:0 | CER 18:0(OH)(2H) | CER 23:0(OH) | CER 28:0 |
| CER 12:1(2H) | CER 18:0 | CER 23:0(OH)(2H) | CER 28:1 |
| CER 13:0 | CER 18:0(2H) | CER 23:0 |  |
| CER 13:0(2H) | CER 18:1 | CER 23:0(2H) |  |
| CER 14:0(OH) | CER 18:1(2H) | CER 23:1 |  |
| CER 14:0 (OH)(2H) | CER 19:0(OH) | CER 23:1(2H) |  |

CER: ceramide. List of ceramides measured in MECHE serum samples
