# Supplementary material for: Uncovering Factors Related to Pancreatic Beta-Cell Function
Source: PLoS One. 2016 Aug 18;11(8):e0161350. doi: 10.1371/journal.pone.0161350 (PMC4990237; doi:10.1371/journal.pone.0161350)
Supplement: S2 Table — Summary of strongest predictors of beta-cell function using linear regression analysis. WHR, waist-to-hip ratio; HDL, high density lipoprotein cholesterol; RA index, resistin-to-adiponectin ratio; cer, ceramide. Data are presented as beta coefficient and P-value according to disposition index, using C-peptide data (nmol mmol-1); beta-cell function using C-peptide (glucose in mmol l-1, c-peptide in nmol l-1) adjusted for the Matsuda index; P-value determined using backward linear regression analysis. Significance level = P < 0.05. Demographic and Anthropometric variables included were: age, sex, BMI, WHR, BP SYS, BP DIA. Biochemical variables included were: HDL cholesterol, adiponectin, resistin, RA index, triacylglycerides, Apo E, TNFα, IFNγ, IL2, IL4, IL6, IL8, IL10. Ceramide data from lipidomic analysis was examined. *RA index in combination with IL-8 was significant predictor of beta-cell function (C-peptide)* Matsuda index using linear regression (p = 0.043). (DOCX) [file pone.0161350.s005.docx]

**Online Supplementary Material**

**S2 Table. Linear regression of anthropometric, biochemical and ceramide data against additional beta-cell function measures**

| **Predictor** | **Disposition index (using C-peptide) (nmol mmol^-1^)** | | **Beta-cell function (C-peptide) *Matsuda index** | |
| --- | --- | --- | --- | --- |
|  | **Beta coefficient** | ***P*** | **Beta coefficient** | ***P*** |
| **WHR** | -0.44 | <0.001 | -0.35 | 0.001 |
| **RA index** | -0.28 | 0.006 | -0.20 | 0.055* |
| **Cer 12:1(2H)** | -0.18 | 0.036 | -0.23 | 0.012 |

Summary of strongest predictors of beta-cell function using linear regression analysis. WHR, waist-to-hip ratio; HDL, high density lipoprotein cholesterol; RA index, resistin-to-adiponectin ratio; cer, ceramide. Data are presented as beta coefficient and P-value according to disposition index, using C-peptide data (nmol mmol^-1^); beta-cell function using C-peptide (glucose in mmol l^-1^, c-peptide in nmol l^-1^) adjusted for the Matsuda index; P-value determined using backward linear regression analysis. Significance level = P < 0.05. Demographic and Anthropometric variables included were: age, sex, BMI, WHR, BP SYS, BP DIA. Biochemical variables included were: HDL cholesterol, adiponectin, resistin, RA index, triacylglycerides, Apo E, TNFα, IFNγ, IL2, IL4, IL6, IL8, IL10. Ceramide data from lipidomic analysis was examined. *****RA index in combination with IL-8 was significant predictor of beta-cell function (C-peptide)* Matsuda index using linear regression (p=0.043).
