# Supplementary material for: Uncovering Factors Related to Pancreatic Beta-Cell Function
Source: PLoS One. 2016 Aug 18;11(8):e0161350. doi: 10.1371/journal.pone.0161350 (PMC4990237; doi:10.1371/journal.pone.0161350)
Supplement: S3 Table — All values are means ± standard deviation. BMI, Body Mass Index; BP SYS, Systolic Blood Pressure; BP DIA, Diastolic Blood Pressure; HOMA-IR, Homeostatic Model Assessment of Insulin Resistance; BCF/HOMA-IR, beta-cell function adjusted by HOMA-IR; BCF*Matsuda index; beta-cell function adjusted by the Matsuda index (where glucose mg dl-1and insulin μIU ml-1) RA index, resistin to adiponectin ratio. (DOCX) [file pone.0161350.s006.docx]

**Online Supplementary Material**

**S3 Table. Baseline characteristics FHI cohort (n=47)**

| Variable | Mean ± S.D. |
| --- | --- |
| Sex (m/f) | 28/19 |
| Age (y) | 53 ± 7 |
| Weight (kg) | 94.30 ± 15.35 |
| BMI (kg m^-2^) | 32.1 ± 4.6 |
| Waist (cm) | 92.69 ± 10.61 |
| BP SYS (mm Hg^-1^) | 127.15 ± 13.69 |
| BP DIA (mm Hg^-1^) | 82.10 ± 8.16 |
| Glucose (mmol l^-1^) | 5.67 ± 0.65 |
| Insulin (µIU ml^-1^) | 12.91 ± 9.79 |
| HOMA IR | 3.23 ± 2.41 |
| BCF/HOMA-IR (pmol mmol^-1^) | 11.83 ± 9.03 |
| Disposition index (pmol mmol^-1^) | 2.83 ± 1.96 |
| BCF*Matsuda index | 9.45 ±7.39 |
| Adiponectin (ug ml^-1^) | 11.75 ± 6.44 |
| Resistin (ng ml^-1^) | 9.14 ± 2.97 |
| RA index | 0.97 ± 0.51 |

All values are means ± standard deviation. BMI, Body Mass Index; BP SYS, Systolic Blood Pressure; BP DIA, Diastolic Blood Pressure; HOMA-IR, Homeostatic Model Assessment of Insulin Resistance; BCF/HOMA IR, beta-cell function adjusted by HOMA-IR; BCF*Matsuda index; beta-cell function adjusted by the Matsuda index (where glucose mg dl^-1^and insulin µIU ml^-1^) RA index, resistin to adiponectin ratio
